# A Narrative Review of the Cases of Co-altered EGFR and ALK in Non-small Cell Lung Cancers: An Attempt to Break the Therapeutic Dilemma

**DOI:** 10.7759/cureus.117425

**Published:** 2026-10-04

**Authors:** Amitabh Kumar Upadhyay, Manoj Kumar, Aaditya Prakash, Jayita Purkait

**Affiliations:** 1 Medical Oncology, Tata Main Hospital, Jamshedpur, IND; 2 Radiation Oncology, Tata Main Hospital, Jamshedpur, IND; 3 Palliative Care, Tata Main Hospital, Jamshedpur, IND

**Keywords:** ca-egfr/alk, co-mutated, eml4-alk, non-small cell lung cancer, tyrosine kinase inhibitors

## Abstract

Molecular profiling is essential in advanced non-small cell lung cancer (NSCLC) to identify actionable genetic alterations and guide targeted therapies. While epidermal growth factor receptor (*EGFR*) mutations and anaplastic lymphoma kinase​​​​​​​ (*ALK*) rearrangements are historically considered mutually exclusive alterations, numerous cases of co-occurring *EGFR* and *ALK* alterations (CA-EGFR/ALK) have been reported. Currently, the clinicopathological profile of this co-altered patient population remains poorly understood, and no established clinical consensus exists regarding optimal treatment sequencing. Our objective was to evaluate the published data related to CA-EGFR/ALK in terms of clinicopathologic features, the pattern of therapy, response to therapy, and survival. This was a retrospective analysis of published literature related to the subject. Relevant case reports, institutional data analyses, review articles, and editorials were searched, and all relevant case reports and research papers with CA-EGFR/ALK were included without any exclusion criteria.

The pooled data of a total of 300 cases showed an Asian and female preponderance with a median age of 57. Smoking history was noted in 22.7%, and adenocarcinoma was the most common histology, with predominant acinar and solid patterns. The most prevalent EGFR mutations were Del 19 and L858R. While response rates to anti-EGFR and ALK therapies have generally been favorable, there are varied results and interpretations. There were no statistical differences in disease control rates (DCR) among EGFR, ALK, or a combination of therapies. Most studies have utilized first-generation anti-EGFR drugs and crizotinib. Moreover, the reported survival outcomes exhibit inconsistencies across the published literature. The key takeaway messages from 41 studies are presented in the table for greater insight.

The largest collective interpretation of the data to date is anticipated to provide deeper insight into the natural history and clinicopathological profile of these cases and help select precision therapies. There exists a clear need for research to identify the preferred anti-EGFR or ALK drugs and the role of combination therapies.

## Introduction and background

Lung cancer (LC) ranks as the second most prevalent malignancy globally, predominantly affecting males and accounting for approximately 2.2 million new cases and 1.8 million fatalities annually [1]. According to the 2020 GLOBOCAN report, it remains the primary driver of cancer mortality. Rising global incidence rates are largely fueled by expanding industrialisation and tobacco consumption [1,2]. Histologically, LC is categorized into two main types: highly aggressive small cell lung cancer (SCLC), representing 15% of cases, and non-small cell lung cancer (NSCLC), which constitutes the remaining 85% [1,2].

NSCLC is further divided into distinct subtypes: lung adenocarcinoma (L-ADC), which is the most common and comprises about 40%; lung squamous cell carcinoma (L-SCC), comprising about 25-30%; and lung large cell carcinoma (L-LCC), making up about 5-10% of cases, alongside rarer subtypes (each accounting for under 2-5%) such as sarcomatoid, adenosquamous, and large cell neuroendocrine carcinomas [2,3]. Because of its aggressive nature and the lack of noticeable symptoms in the early stages, LC is often diagnosed when the disease has significantly advanced. It can present in various ways, including localized tumor effects, regional or distant spread, or paraneoplastic syndromes [4]. Advanced diagnostic techniques have led to more precise pathological and genomic classification, enabling more targeted therapeutic interventions. The fifth edition of the lung cancer classification framework integrates advanced molecular testing and immunohistochemistry (IHC) techniques, enhancing the accuracy of both genomic and pathological profiling. Therapeutically, surgical resection remains the definitive standard of care for early-stage NSCLC, whereas systemic chemotherapy is typically utilized for patients presenting with advanced disease [5].

Molecular profiling is vital for treating advanced NSCLC patients who benefit from precision targeted therapies. Approximately 60% of L-ADC cases harbor an identifiable, oncogenic driver mutation that kickstarts tumor development. According to updated clinical NCCN Guidelines, standard comprehensive biomarker panels screen for core mutations, including KRAS (15-25%, driven heavily by the targetable KRAS G12C variant), epidermal growth factor receptor (EGFR) (10-15% in Western and up to 40-50% in Asian populations), anaplastic lymphoma kinase​​​​​​​ (ALK) fusions (3-7%), MET exon 14 skipping (3-4%), BRAF V600E (1-3%), and PIK3CA (2-4%). This panel also encompasses lower-frequency, yet highly actionable alterations such as ROS1 fusions (1-2%), HER2 (ERBB2) mutations (1-3%, primarily driven by exon 20 insertions), RET fusions (1-2%), NTRK1/2/3 fusions (<1%), and de novo or acquired MET amplifications (1-3%). Clinical practice additionally tests for critical newer and rare biomarkers, such as NRG1 fusions (<1%), distinct EGFR exon 20 insertions (~2%), TROP-2 surface expression, and immuno-resistance co-mutation signatures like STK11 and KEAP1 [6,7]. SCLC accounts for roughly 13-15% of cases and is an aggressive neuroendocrine tumor strongly linked to smoking. Driven by universal TP53 and RB1 loss, its frontline treatment relies on chemoimmunotherapy, while advanced options include targeted therapies like the DLL3-directed bispecific T-cell engager tarlatamab [7,8].

EGFR gene

The EGFR gene, mapped to chromosome 7, codes for a cell-surface receptor tyrosine kinase (RTK) routinely found on most healthy cells [8,9]. Structurally, this receptor spans three segments: an extracellular ligand-binding domain, a hydrophobic transmembrane region, and an intracellular domain featuring tyrosine kinase activity. Interaction between the RTK and various EGF receptor ligands initiates receptor activation and subsequent autophosphorylation of its cytoplasmic domain. This molecular event triggers vital downstream signal transduction cascades, including the PI3K-AKT and Ras/Raf/mitogen-activated protein kinase (MAPK) pathways. These pathways regulate tumor proliferation, invasion, resistance to apoptosis, and neo-angiogenesis [8-10].

In NSCLC, mutations occur almost exclusively in the exons encoding the kinase domain (exons 18 to 21), with the most common mutations being the in-frame deletion of exon 19 (del19) and the L858R mutation in exon 21 [9-12]. Del19 accounts for 44-51%, and the exon 21 L858R mutation accounts for 38-40% of EGFR mutations in NSCLC [9-12]. Sanger sequencing is limited by low sensitivity (15-20%) and high tumor cellularity demands (≥50%). Reverse transcription polymerase chain reaction (RT-PCR) and droplet digital PCR (ddPCR) provide sensitive, single-gene targeted testing validated for NSCLC tissue and liquid biopsies. Conversely, next-generation sequencing (NGS) simultaneously detects multiple concurrent mutations and tyrosine kinase inhibitor (TKI) resistance mechanisms; it excels with scarce tissue but is constrained by high costs and long turnaround times [13-15].

Three generations of EGFR TKIs treat EGFR-mutant NSCLC: first-generation (gefitinib, erlotinib, icotinib), second-generation (afatinib, dacomitinib), and third-generation (osimertinib, lazertinib). Third-generation agents effectively bypass acquired T790M mutations and optimize survival. To overcome subsequent resistance (like the C797S mutation), fourth-generation TKIs (e.g., silevertinib, JIN-A02) are currently under clinical development [10].

ALK gene 

The ALK gene on chromosome 2 drives tumors like NSCLC via rearrangements (ALK-R, mostly EML4-ALK in 3-7% of cases), amplifications (ALK-A), and point mutations. ALK-R is mutually exclusive with EGFR or KRAS mutations [16-18]. Diagnosed via fluorescence in situ hybridization (FISH; gold standard), IHC (routine), or RT-PCR, advanced ALK-R NSCLC survival has vastly improved via targeted ALK inhibitors, with trials exploring novel fourth-generation inhibitors [6,7,16-18].

L-ADC mutational epidemiology depends on demographic and environmental factors. Never-smokers and females skew toward EGFR mutations, while ROS1, ALK, and RET fusions affect young patients regardless of gender. Heavy smoking correlates with KRAS, NRAS, MAP2K1, and TP53 mutations; MET and BRAF span both cohorts. EGFR mutation incidence is lower in Western/African-American cohorts (10%-15%) but significantly higher in Asians, peaking at 50.5% in Taiwanese populations [19-22]. ALK-R patients are typically younger light/non-smokers, histologically presenting solid growth with signet ring cells or cribriform/mucinous architectures [16-18]. Targeted LC therapies revolutionized treatment, but resistance usually develops within a year. Mapping targetable alterations alongside resistance mechanisms is critical to improving management and alternative therapies [23].

Co-altered EGFR and ALK

In the context of NSCLC, the presence of the ALK-R has been associated with a wild-type EGFR status. However, in 2008, Koivunen et al. made a groundbreaking discovery by identifying the co-occurrence of the EGFR mutation and the EML4-ALK fusion (CA-EGFR/ALK) during a study on the sensitivity of three NSCLC cell lines to ALK inhibitors [24]. This finding significantly contributed to the understanding of co-mutations and led to the subsequent publications of various aspects of this phenomenon. Research has highlighted the significance of evaluating co-occurring mutations and studying tumor heterogeneity to understand the tumor molecular landscape comprehensively, which has implications for treatment responses [25]. In cases of CA-EGFR/ALK, EGFR and ALK may engage in bypass signaling, resulting in poor responses to TKIs targeting both pathways. Another potential mechanism involves the intratumoral expression of the EGFR mutation and rearrangement in separate cells [25].

Objective

To date, there have been many reported cases of CA-EGFR/ALK, encompassing institutional data, regional data, case series, and case reports. Some papers have discussed and analyzed 97 cases [25]. However, there is limited consensus regarding its clinicopathological profile and no established consensus on the treatment sequencing for these rare occurrences. Our objective was to evaluate the published data related to CA-EGFR/ALK in terms of clinicopathologic features, the pattern of therapy, response to therapy, and survival. A pooled analysis of the existing literature will likely provide valuable insights for clinicians managing such cases.

Materials and methods

This was a narrative review with retrospective analysis of published literature related to the subject. 

Methodology

Relevant case reports, institutional data analyses, review articles, and editorials were searched on PubMed, Embase, Cochrane Central Register, Web of Science Core Collection, Scopus database, and Google search engine. Keywords like "synchronous EGFR and ALK mutations, concomitant, co-mutation, epidermal growth factor receptor mutation and anaplastic lymphoma kinase translocation together, rare double mutations EGFR and ALK" were used. All relevant case reports and research papers with CA-EGFR/ALK were included, irrespective of year of publication and language. There were no exclusion criteria. After reviewing the selected articles' reference list, other relevant articles were searched and included. 

Statistics

The study did not include a sample size calculation. All patients diagnosed with CA-EGFR/ALK were included in the study. According to the protocol, data from the papers were entered into Excel (Microsoft, Redmond, WA, USA). The details recorded included the year of publication, demographics, smoking status, clinicopathological profile, mutation profile, and treatment details, mainly focusing on anti-EGFR and ALK therapies. The data were analyzed using SPSS Statistics Version 23.0 (IBM Corp., Armonk, NY, USA). Fisher's exact test was used to check for statistically significant differences between disease-controlled rates (DCR) as per assumptions.

## Review

We identified 41 case reports/series and 41 papers containing institutional/regional data, totalling 300 cases [24,26-104].

The combined data for CA-EGFR/ALK revealed a higher number of female cases, with 103 males and 141 females, resulting in a male-to-female ratio of 0.73. The majority of reported patients were of Asian descent. The median age was 57 years, with 45 (22.7%) patients having a smoking history. ADC was the most common histology, accounting for 254 cases, primarily showing acinar and solid patterns, and around 81.6% of patients had documented metastatic disease. Disease burden in metastatic cases was available for 85 cases. The complete demographic and clinicopathological summary of the cases is presented in Table 1.

**Table 1:** Demographic and clinicopathological summary NSCLC: non-small cell lung cancer, NOS: not otherwise specified

Parameter	Data available	Number (%)
Total number of cases	51 from case reports/series + 249 from studies	300
Sex distribution	244	Males: 103 (42.2) Females: 141 (57.8)
Median age	251/184	59/57 years Males: 56 years Females: 60 years
Stage distribution	229	Stage I: 10 (4.3) Stage II: 6 (2.6) Stage III: 26 (11.3) Stage IV: 187 (81.6)
Smoking	197	Smokers: 45 (22.8) Nonsmokers: 152 (77.2)
Metastatic disease burden	85	Brain: 35 (41.1) Bone: 28 (32.9) Pleural effusion: 16 (18.8) Non-regional lymph nodes: 15 (17.6) Lung: 8 (9.4) Liver: 8 (9.4) Adrenal: 6 (7.1) Intrathoracic metastasis: 19 (22.3) Intra-abdominal metastasis: 13 (15.2)
Histology	260	Adenocarcinoma: 254 (97.6) NSCLC NOS: 4 (1.5) Adenosquamous: 2 (0.7)
Histological subtype	121	Solid: 26 (21.4) Acinar: 22 (18.9) Papillary: 9 (7.4) Signet ring cell: 9 (7.4) Cribriform: 5 (4.1) Mucinous: 4 (3.3) Micropapillary: 3 (2.4) Lepidic: 2 (1.6) Bronchoalveolar: 1 (0.8)
Documented intent of treatment	233	Curative: 35 (15) Palliative: 198 (85)

Details of EGFR mutations were accessible in 250 cases, with the most prevalent mutations being Del 19 and exon 21 L858R. There were 12 cases with compound or complex EGFR mutations. The spectrum of EGFR mutations can be found in Table 2.

**Table 2:** Spectrum of EGFR mutations

Exon	Number	Mutation	Number
Exon 18	8	Exon 18 (G719A)	1
		Exon 18 (G719X)	6
		Exon 18 (L718P)	1
Exon 19	139	Del 19	139
Exon 20	10	Exon 20 (R803W)	2
		Exon 20 (V769M)	1
		Exon 20 (T790M)	3
		Exon 20 insertion	4
Exon 21	80	Exon 21 (L858A)	1
		Exon 21 (L858Q)	1
		Exon 21 (L858R)	66
		Exon 21 (L861Q)	9
		Exon 21 (L862R)	1
		Exon 21 (G863D)	1
		Exon 21 (L868K)	1
Exon 23	1	Exon 23 polymorphism	1
Compound/Complex	12	Exon 18 (G719A) + Exon 19 (L747V)	1
		Exon 18 (G719C) + Exon 20 (S768I)	1
		Exon 18 (G719X) + Exon 21 (L858R)	1
		Exon 18 (G719X) + Exon 21 (L861Q)	1
		Del 19 + Exon 20 (T790M)	1
		Del 19 + Exon 20 insertion	3
		Del 19 + Exon 21 (L858R)	1
		Exon 20 (S811P, L782F)	1
		Exon 21 (L858R) + T790M	1
		Exon 21 (L858R) + Exon 21 (L833V)	1

A total of 156 cases received anti-EGFR therapy as first-line TKI, 25 received ALK therapy, and nine received a combination of EGFR and ALK therapy. Best treatment responses were documented in 149 cases for EGFR therapy, 22 for ALK therapy, and nine for combination therapy. DCRs with first-line EGFR therapy versus ALK therapy vs. combination EGFR and ALK therapy were 76.5%, 84.2%, and 88.8%, respectively. Using Fisher's exact test at a 5% significance level, we found no significant difference (p=0.5606).

Following failure with first-line TKIs, 58 patients received ALK TKIs, five received EGFR TKIs, and eight received a combination of TKIs. Best response data were documented for 58 patients on ALK TKIs, five on EGFR TKIs, and seven on a combination of TKIs. DCRs with second-line EGFR therapy versus ALK therapy versus combination EGFR and ALK therapy were 60, 72.4, and 100%, respectively. Using Fisher's exact test at a 5% significance level, we found no significant difference (p=0.2194).

Progression-free survival (PFS) data for first-line treatment were documented for 139 patients receiving EGFR TKIs, 20 receiving ALK TKIs, and nine receiving a combination of TKIs. Similarly, PFS data for second-line treatment was documented for 53 cases with ALK therapy, five with EGFR therapy, and eight with a combination of EGFR and ALK therapies. Overall survival (OS) data was reported for 82 cases; 28 patients received only EGFR therapy, five received only ALK therapy, and 49 received both EGFR and ALK therapies. As many patients were still undergoing treatment with TKIs at the time of data publication, the median PFS or OS could not be calculated.

Table 3 describes the key messages from 41 studies related to CA-EGFR/ALK.

**Table 3:** Key messages from 41 studies related to co-altered epidermal growth factor receptor/anaplastic lymphoma kinase (CA-EGFR/ALK) ALK: anaplastic lymphoma kinase, ALK-R: ALK rearrangement, EGFR: epidermal growth factor receptor, EML4: echinoderm microtubule-associated protein-like 4, EML4-ALK: echinoderm microtubule-associated protein-like 4 and anaplastic lymphoma kinase fusion, FISH: fluorescence in situ hybridization, GGN: ground-glass nodule, IHC: immunohistochemistry, L-ADC: lung adenocarcinoma, NGS: next-generation sequencing, NSCLC: non-small cell lung cancer, ORR: objective response rate, OS: overall survival, PCR: polymerase chain reaction, PD: progressive disease, PR: partial response, PFS: progression-free survival, RET: rearranged during transfection, RT-PCR: reverse transcription polymerase chain reaction, SCLC: small cell lung cancer, SD: stable disease, TKI: tyrosine kinase inhibitor

Author	Key learnings
Koivunen P [24]	Identified the co-occurrence of the EGFR mutation and the EML4-ALK fusion (CA-EGFR/ALK) during a study on the sensitivity of three NSCLC cell lines to ALK inhibitors.
Zhang X [65]	Investigators used PCR sequencing on 103 NSCLC patients to map ALK fusions. They identified EML4-ALK fusions in 11.6% of the cohort (12 patients), noting higher prevalences in specific subgroups: 16.13% in L-ADC, 19.23% in lifetime non-smokers, and 42.80% in EGFR/KRAS wild-type L-ADC. The presence of the fusion correlated significantly with youth and a non-smoking history. Notably, the authors also detailed the first Chinese female L-ADC patient presenting with co-occurring EGFR/ALK alterations.
Camidge DR [66]	Researchers screened 73 patients using FISH to assess intratumoral heterogeneity, uncovering 13 ALK-positive cases. The cohort exhibited typical rearrangements coupled with gene copy number gains. Notably, all positive cases were restricted to L-ADC, and the marker appeared in 44.8% of EGFR/KRAS wild-type patients with a light smoking history (<10 pack-years). FISH analyses demonstrated distinct variation in ALK positivity, yielding significantly greater mean signals in the tumor regions of positive patients than in normal adjacent tissue or negative control samples. Counting 60 or more cells across at least four fields optimized the protocol to yield perfect 100% sensitivity and specificity.
Sasaki T [67]	Sasaki et al. found that the activation of EGFR signaling was a bypass signaling mechanism contributing to ALK inhibitor resistance.
Yang J [68]	Found that 10.6% (42 out of 398) of NSCLC patients had the EML4-ALK fusion, while CA-EGFR/ALK were present in 1.0% (4 out of 398) of NSCLC patients. All of these patients were never smokers, had L-ADC, and had wild-type KRAS. Two female cases with CA-EGFR/ALK received first-line gefitinib and achieved a PR. Further investigation is needed to determine if they could also benefit from ALK inhibition after failing to respond to EGFR TKIs.
Wang J [69]	The incidence of ALK mutation was 9.7%. Patients with CA-EGFR/ALK showed similar ORRs after EGFR-TKI, although inferior PFS compared to those with isolated EGFR mutation.
Lee JK [70]	Screening 444 Korean L-ADC patients revealed an EGFR mutation rate of 51.4% and an ALK rearrangement rate of 7.7%. The study also identified four patients (0.9%) with co-existing EGFR/ALK alterations, three of whom underwent curative surgeries. Intriguingly, when one patient received separate lines of EGFR and ALK inhibitors, they only responded to the ALK-targeted therapy. This divergence suggests that dual-positive patients vary in their oncogenic reliance, meaning they may require distinct, individualized targeted treatment strategies.
Boland JM [71]	Discussed one of 25 ALK-positive cases showing EGFR mutation.
Xia N [72]	Reported two female non-smoking patients harboring CA-EGFR/ALK out of 110 patients.
Wang J [73]	Reported the frequency of the EGFR or the KRAS mutations and the ALK-R as 44.9%, 7.2%, and 9.6%, respectively, in a study of 332 patients. Only one (1/332, 0.3%) patient had CA-EGFR/ALK.
Won JK [74]	Screening 1,458 NSCLC cases demonstrated that using advanced, high-sensitivity tools like RNA RT-PCR and NGS ultra-deep sequencing increased CA-EGFR/ALK detection rates to 8.8%, 12.1%, and 15.4%, respectively. Investigators noted poor outcomes in three dual-positive patients who received the EGFR inhibitor gefitinib. In contrast, ALK inhibitors (crizotinib or ceritinib) yielded excellent results in eight patients, achieving an 87.5% response rate (7/8 partial responses) and extended PFS. Tumour heterogeneity influenced gefitinib efficacy, with patient outcomes varying according to the abundance of EGFR-mutant cells. Varying loads of ALK-positive cells likewise dictated how well individual patients responded to crizotinib therapy.
Yang JJ [75]	The authors evaluated 977 NSCLC patients for both ALK rearrangements and EGFR mutations, identifying a 1.3% (13/977) frequency of co-occurring alterations. First-line EGFR-TKI therapy induced a partial response in 80% (8/10) of treated tumours, yielding a median PFS of 11.2 months. Using IHC, investigators demonstrated that mutant EGFR and ALK fusion proteins were co-expressed within the same tumor cells. The study concluded that measuring phospho-ALK and phospho-EGFR ratios can successfully forecast how well patients respond to crizotinib and EGFR-TKIs. Heterogeneous responses to initial EGFR-TKIs matched the tumors' phospho-EGFR levels, whereas phospho-ALK abundance dictated the success of later crizotinib treatment.
Cabillic F [76]	Concurrent IHC and FISH testing for ALK rearrangements across a cohort of 3,244 consecutive NSCLC cases revealed that 150 patients (4.6%) tested positive by at least one modality. Concordance between the two methods was limited to 80 of the 150 positive cases, indicating that relying on a single assay would have failed to detect approximately 25% of the ALK-positive population. This diagnostic divergence emphasizes the clinical necessity of a dual-testing strategy to accurately identify candidates for ALK inhibitors, particularly given that a subset of patients with discordant results still derived benefit from crizotinib.
Kim TJ [77]	Simultaneous panel screening for EGFR, KRAS mutations, and ALK-R was conducted on 510 NSCLC Korean patients. The results showed that simultaneous genotyping identified EGFR mutations in 29.0% of the patients, KRAS mutations in 8.6%, and ALK-R in 9.2%. Additionally, seven patients had overlapping mutations, including five with CA-EGFR/ALK. The study found no relationship between ALK rearrangement and the patients' sex or smoking history.
Marino FZ [78]	Intratumoral heterogeneity dynamics regarding EGFR mutations and ALK rearrangements were analyzed within a cohort comprising 105 mixed L-ADC and 17 adenosquamous carcinomas. Among the 122 total cases, ALK rearrangements were detected in 10 tumors, with 70% (7/10) demonstrating distinct intratumoral heterogeneity. Conversely, EGFR mutations were identified in 12 cases, all of which exhibited uniform mutant protein expression across every histological component. These data indicate that EGFR mutations are typically distributed homogeneously throughout the tumor matrix. In contrast, ALK rearrangements frequently manifest intratumoral heterogeneity in both mixed L-ADC and adenosquamous subtypes, a factor that may influence clinical responses to targeted therapy.
Jhu J [79]	Analyzed 131 Chinese patients with L-ADC for mutations in the EGFR, KRAS, and ALK-R. Two never-smoking women carried a CA-EGFR/ALK.
Xu L [80]	Identified three CA-EGFR/ALK cases in never-smoking females.
Cai W [81]	Researchers analyzed how intratumoral heterogeneity affects the genomic and pathological traits of ALK-rearranged L-ADC in 629 patients. The screening revealed 30 ALK-positive cases (4.8%), 364 EGFR-mutant cases (57.9%), and two patients with dual CA-EGFR/ALK alterations. Using RT-PCR, the team successfully mapped intratumoral ALK fusion variations in nine individuals. Crucially, the two dual-altered patients exhibited distinct genomic heterogeneity, which manifested both across different tumor growth patterns and within single, uniform growth patterns.
Wu C [82]	Investigators analyzed discrepancy rates among eight primary driver mutations in matched, multiple intrapulmonary GGNs from NSCLC patients. The team evaluated 72 total lesions (including 60 GGNs) from 35 patients who had undergone resections for multiple tumors featuring confirmed NSCLC and at least one GGN. They screened for mutations in EGFR, KRAS, HER2, BRAF, and PIK3CA, plus ALK, ROS1, and RET fusions. Comparing matched tumors revealed that just six out of 30 patients shared identical genetic profiles across lesions, demonstrating a striking 80% driver mutation discordance rate among mutation-positive individuals.
Sholl LM [83]	The Lung Cancer Mutation Consortium was created to streamline multi-center, collaborative evaluations tracking 10 distinct, actionable oncogenic driver alterations. Among 1007 patients from 14 institutions, EGFR, KRAS, ALK, and ERBB2 alterations were found in 22%, 25%, 8.5%, and 2.4% of cases, respectively. Additionally, there were three cases with CA-EGFR/ALK.
Kim EY [84]	Investigators studied the clinical impact of complex EGFR mutations by running a 16-gene NGS-based recurrent deep sequencing panel on 61 patients with EGFR-positive L-ADC. Compound EGFR mutations were detected in 15 (24.6%). Most compound mutations (12 out of 15) were comprised of combinations of atypical and typical mutations, such as exon 19 deletion, L858R or G719X substitutions, or exon 20 insertion. Three of these compound mutations consisted of rare atypical mutations. Patients with compound mutations exhibited a significantly shorter OS compared to those with simple mutations (83.7 vs. 72.8 months; P = 0.020). The study also revealed five cases of CA-EGFR/ALK. The cases with compound mutations showed a higher burden of missense mutations.
Lou NN [85]	The authors tracked clinical success and TKI resistance mechanisms across 118 advanced NSCLC cases positive for either EGFR or ALK. Initial EGFR-TKI therapy succeeded in 90% of CA-EGFR/ALK patients. Meanwhile, crizotinib treatment in isolated ALK-rearranged cases showed a 40% response rate and 1.9-month median PFS, underperforming compared to the 73.9% response rate and 6.9-month median PFS seen in dual-altered patients. Median survival spans reached 21.3 months for the EGFR group, 23.7 months for the ALK group, and 18.5 months for the CA-EGFR/ALK group, showing a significant statistical difference in survival between the ALK and dual-mutation patients. The study pinpointed multiple resistance pathways, including increased phospho-EGFR levels during crizotinib therapy, acquired *EGFR* T790M mutations, and histological transformation into SCLC. The findings suggest utilizing frontline EGFR-TKIs for dual-positive cases, while using relative phospho-EGFR and phospho-ALK levels to time sequential crizotinib therapy.
Ulivi P [86]	The authors evaluated 380 NSCLC cases using independent testing streams to detect EGFR, KRAS, and ALK driver alterations. The analysis revealed overlapping mutations, including a 1.6% rate for CA-EGFR/ALK, 1.1% for EGFR/KRAS, and 2.5% for co-existing ALK and KRAS alterations. Frontline TKI therapy benefited 81.8% of the 28 EGFR-mutant patients who had isolated mutations, but that benefit dropped to 67% for patients with co-occurring ALK rearrangements. Notably, one dual-positive CA-EGFR/ALK patient given crizotinib therapy developed PD in under three months.
Lee T [87]	Investigators discovered overlapping mutations in just 12 out of 6,637 screened NSCLC patients, all of whom had *EGFR*-mutant adenocarcinoma. The dual-positive group included six patients with *KRAS* co-mutations and six with ALK rearrangements. Morphologically, the six CA-EGFR/ALK tumors featured solid or cribriform architectures, and half (three patients) showed signet ring cell features. Clinicians treated four CA-EGFR/ALK patients with an *EGFR* TKI and one with an ALK inhibitor. Four of these five individuals achieved a PR or SD, while one patient failed to respond to erlotinib.
Schmid S [88]	Researchers evaluated 14 L-ADC patients featuring combined ALK rearrangements and *EGFR* or *KRAS* mutations, identifying five CA-EGFR/ALK cases and nine ALK/*KRAS* co-alterations. The data revealed that 86% of patients with simultaneous ALK and *KRAS* alterations failed to respond favorably to crizotinib therapy. Conversely, *EGFR* TKI therapy achieved positive results in three out of four ALK/*EGFR* dual-positive patients across different treatment lines, resulting in a median PFS of 5.8 months. The study suggests that patients with CA-EGFR/ALK may have worse outcomes with ALK and EGFR TKIs compared to those with either alteration alone. However, further research is needed to determine which patients with CA-EGFR/ALK may still benefit from the respective TKI.
Sahnane N [89]	Aimed to identify co-altered cases in 42 therapy-naive ALK-R L-ADC. Three patients showed a concurrent mutation in the EGFR, and five cases exhibited a concomitant KRAS mutation. The study found that the two CA-EGFR/ALK patients responded better to crizotinib than erlotinib, while the two KRAS-mutant patients experienced poor responses to crizotinib. The study indicated that crizotinib offers superior efficacy over erlotinib for patients with CA-EGFR/ALK, though the impact of targeted therapies on dual ALK/KRAS mutations requires further clarification.
Lee B [90]	A retrospective study of patients in Korea identified 12 EGFR-mutated tumors with additional mutations in KRAS (6, 0.1%) or ALK (6, 0.1%).
Hu W [91]	Conducted a study on 320 EGFR-mutant NSCLC patients to examine the impact of simultaneous genomic changes on the efficacy of EGFR-TKI treatment. They discovered that 6.6% of patients had additional gene alterations, such as PIK3CA mutation (9 cases), ALK-R (6 cases), HER2 mutation (3 cases), RET rearrangement (1 case), ROS1 rearrangement (1 case), and KRAS mutation (1 case). The data showed that patients with lone EGFR mutations achieved a notably longer PFS than those with co-existing gene alterations (10.9 vs. 6.0 months, P = 0.002). Dual ALK/EGFR positive patients experienced a median PFS of 5.0 months on EGFR TKIs, though crizotinib induced a PR in three out of six patients after initial EGFR TKI therapy failed. The team found no significant statistical difference in OS between the isolated EGFR group and the co-mutated group (21.0 vs. 17.6 months, P = 0.17). Ultimately, the study established that overlapping genetic alterations drastically reduce how well patients respond to EGFR TKIs.
Guibert N [92]	Biomarkers France trial evaluated 17,664 patients to map out the prevalence, clinical ties, prognostic outcomes, and treatment efficacy of multiple simultaneous mutations.Investigators discovered 162 dual-mutant and three triple-mutant cases, finding that *KRAS* (67.3%), *PIK3CA* (53.3%), and *EGFR* (42.4%) were the most prominent co-alterations, while ALK rearrangements appeared in only 10 cases (6%). Concomitant mutations in ALK-positive patients failed to trigger a significant drop in either OS (17.7 vs. 20.3 months, *p* = 0.57) or median PFS (10.3 vs. 12.1 months, *p* = 0.93).
Mao Y [93]	Conducted a study on 421 patients with EGFR mutations who were treated with EGFR-TKIs. The study also looked at the presence of ALK and ROS1 fusion genes and their impact on treatment outcomes. Of the patients, 10 showed ALK positivity, and three showed ROS1 positivity. All patients received EGFR-TKIs, and eight received subsequent ALK/ROS1 inhibitors. The study found that PFS was longer in patients with only EGFR mutations (10.7 months) than those with CA-EGFR/ALK or EGFR/ROS1 mutations (6.6 months, P=0.004). The data indicates no significant difference in OS among the analysed groups. For patients treated with ALK/ROS1 inhibitors, the median PFS was recorded at 6.0 months.
Martín Martorell P [94]	Reported two cases of CA-EGFR/ALK out of 326 patients with nonsquamous NSCLC.
Zhao Y [95]	Investigators compared targeted therapies in patients with CA-EGFR/ALK against those with only a single driver mutation. From a screened population of 5,816 patients, the study tracked 26 CA-EGFR/ALK cases (22 included in survival analysis), alongside a random sample of 95 *EGFR*-mutant and 60 *ALK*-rearranged patients. The ORR for *EGFR* TKIs was essentially identical between the dual-altered group (63.2%) and the *EGFR*-only group (62.1%, *p* = 0.93), with a median PFS of 10.3 versus 11.4 months (*p* = 0.87). Crizotinib achieved very similar success across arms, inducing a 66.7% ORR in dual-positive patients compared to 65.0% in *ALK*-only patients (*p* = 1.00), with a median PFS of 11.1 and 12.5 months (*p* = 0.28). Median overall survival spanned 27.1 months for the *EGFR* arm, 36.2 months for the *ALK* arm, and 36.8 months for the CA-EGFR/ALK group. Dual-positive patients experienced a modest survival advantage over the lone *EGFR*-mutant group, though this difference failed to achieve standard statistical significance (*p* = 0.12). Using crizotinib sequentially after an initial *EGFR* TKI failed yielded a 15.0-month median PFS, which performed just as well as upfront crizotinib use in *ALK*-rearranged patients (*p* = 0.80). Because both first-generation *EGFR* TKIs and crizotinib showed clear efficacy, the authors recommend utilizing sequential inhibitor therapy to manage these dual-mutation cases.
Fan J [96]	The authors compared the genetic and pathological profiles of multifocal and unifocal L-ADCs featuring joint *EGFR/ALK* alterations. They screened 1,059 patients who underwent surgical resections for L-ADC to establish baseline *EGFR* and *ALK* molecular statuses. The analysis revealed that co-occurring alterations were significantly more common in multifocal L-ADC than in unifocal cases, showing a rate of 4.71% (4/97) compared to just 0.83% (8/962). The findings highlight why clinicians must accurately distinguish between synchronous primary tumors and internal metastases, while also measuring the specific mutational loads of both *EGFR* and *ALK*.
Liu J [97]	Investigators analysed 419 ALK-positive NSCLC cases to map molecular traits, discovering a 5.01% (21/419) rate of overlapping CA-EGFR/ALK. The team noted a sharp contrast in EGFR mutation rates, which were significantly lower in patients with *EML4-ALK* fusions (3.06%) than in those with non-*EML4-ALK* partners (16.67%, *p* < 0.01). Gender-based differences appeared, showing that *EML4-ALK*/EGFR mutations favoured female patients, while non-*EML4-ALK*/EGFR variations occurred more often in males (*p* = 0.02). When given an EGFR-TKI, the *EML4-ALK*/EGFR group (n = 7) experienced a dramatically reduced median PFS compared to the non-*EML4-ALK*/EGFR group (n = 8; 6.0 vs. 15.0 months, *p* = 0.046). Single-agent TKI strategies produced highly inconsistent clinical responses among five CA-EGFR/ALK patients. In contrast, all three patients who received a dual-TKI combination (EGFR-TKI plus ALK-TKI) successfully pushed their PFS beyond the five-month mark (8.4, 8.6, and >5.2 months). Overall, the two co-alteration subtypes demonstrated completely different clinical behaviour and responses to single-target EGFR therapies. This divergence suggests that non-*EML4-ALK* combinations might develop as a protective resistance mechanism against EGFR-TKIs. Ultimately, the trial highlights dual-TKI combinations as a far more practical and powerful treatment option than single-agent protocols.
Zhuang X [98]	A large-scale molecular analysis was conducted on 3,774 tumor specimens from patients with NSCLC to evaluate alterations across the EGFR, ALK, ROS1, KRAS, and BRAF oncogenes. The screening identified overlapping driver mutations in a small subset representing 1.7% of the total cohort, which translated to 63 individual positive samples. Frontline therapeutic intervention with an EGFR TKI failed to demonstrate a statistically significant expansion in PFS among individuals harbouring co-occurring EGFR mutations relative to those presenting with an isolated EGFR driver.
Mohapatra PR [99]	Investigators screened 198 L-ADC specimens for both EGFR and ALK markers, discovering four male patients with advanced-stage CA-EGFR/ALK. The team observed a clear age split in mutations: younger patients presented with EGFR exon 19 deletions, while older patients carried exon 21 mutations. Frontline EGFR TKI therapy achieved solid disease control at the six-month mark, inducing a PR in two patients and SD in the other two.
Yang X [100]	Investigators reviewed 271 ALK-positive and 2,975 EGFR-positive patients, uncovering nine rare cases (2.6% of the ALK group and 0.2% of the EGFR group) with dual CA-EGFR/ALK. The medical team treated eight of these dual-positive individuals with at least one line of EGFR TKI therapy. Patients achieved strong clinical results on first-generation EGFR TKIs, reaching a median PFS of 14.5 months. Because no patient carried ALK mutations before starting therapy, the authors suggested these mutations likely developed to drive secondary resistance against EGFR inhibitors. Notably, the presence of EGFR/ALK L1152R co-mutations predicted a much shorter PFS during EGFR TKI therapy compared to other genetic combinations.
Zhao Y [101]	Investigators analysed molecular profiles from 3,077 NSCLC patients, finding that roughly 1.5% (46 cases) carried multiple actionable mutations simultaneously, including two rare instances of co-occurring CA-EGFR/ALK.
Li J [102]	Studied 96 patients with IHC-identified ALK fusions for co-alterations and found one with CA-EGFR/ALK.
Noronha V [103]	A large Indian study tracked 24 patients with co-occurring CA-EGFR/ALK, whose initial treatments varied from platinum-based chemotherapy to oral EGFR inhibitors, oral ALK inhibitors, or combination TKI therapy. During their long-term care, 14 patients received both EGFR and ALK inhibitors at some stage, while 10 patients received only one type of targeted drug or none at all. At a median follow-up of 32 months, patients who had access to both EGFR and ALK inhibitors achieved a median OS of 39.2 months, compared to just 7.5 months for those restricted to one or neither targeted drug. Adding an ALK inhibitor to the treatment plan significantly extended patient survival, and severe (grade ≥3) side effects were highly similar across all groups.
Zhong WX [104]	Among 237 L-ADC patients harbouring EGFR mutations or ALK rearrangements, six cases (2.53%) exhibited EGFR/ALK co-alterations. This dual-mutation profile correlated with female sex, non-smoking status, and stage IV disease featuring bone metastasis. Managed primarily with EGFR TKIs, these patients achieved a PFS of two to six months.

Won et al. showed that the detection rate of CA-EGFR/ALK in NSCLC patients increased when sensitive detection methods for EGFR mutation, such as peptide nucleic acid (PNA) RT-PCR and ultra-deep sequencing by NGS, were used [74]. Similarly, Cabilic et al. reported parallel FISH and IHC for ALK-R testing on NSCLC cases. They found that in 150 cases (4.6%), either FISH or IHC yielded a positive result. Surprisingly, both methods only identified 80 out of the 150 cases as ALK-positive. The authors concluded that relying on a single FISH or IHC analysis would have missed about one-fourth of ALK-positive cases. These discrepant results emphasize that combined testing is essential to accurately identify patients eligible for ALK inhibitors, particularly as some with discordant profiles still benefit from crizotinib [76]. Additionally, the prevalence of EGFR mutations within our dual CA-EGFR/ALK cohort aligned with rates typically observed in isolated EGFR-mutated NSCLC.

CA-EGFR/ALK in NSCLC has gained attention, but we still lack a complete understanding of the underlying biological mechanisms. The high histological and molecular heterogeneity of NSCLC may reflect a link between tumor appearance and molecular diversity. Distinct cellular characteristics shape these co-mutations, as Marino et al. demonstrated that EGFR mutations are generally homogeneously expressed, whereas ALK rearrangements exhibit intratumor heterogeneity that can impact therapeutic responses [78]. This ALK-R heterogeneity manifests across and within different growth patterns, and has been confirmed via FISH. Clinically, Fan et al. reported a significantly higher prevalence of CA-EGFR/ALK in multifocal L-ADC than in unifocal cases [96]. Two primary hypotheses explain the underlying biological mechanisms of these co-mutations: a polyclonal origin involving two distinct cell lines harboring separate alterations within the same tissue, or a monoclonal origin in which a single cell population simultaneously carries both EGFR mutations and ALK rearrangements. This complex tumor heterogeneity poses significant challenges for optimal treatment of co-mutated NSCLC [105].

The dominance of oncogenic alterations can vary based on mutation-driven tumor burden and the phosphorylation of single-mutant proteins. The incomplete representation of mutation burden in small biopsies and cytology may explain inconsistent responses to TKIs. Studies have found a positive correlation between the abundance of EGFR mutations at baseline and EGFR TKI effectiveness. Protein phosphorylation levels also heavily influence therapeutic outcomes, as associations exist between EGFR or ALK phosphorylation and TKI efficacy. For instance, Won et al. demonstrated that TKI responses vary depending on the cellular percentage of EGFR mutations and ALK rearrangements [74]. Utilizing IHC, Yang et al. confirmed the co-expression of mutant EGFR and ALK fusion proteins within identical tumor cell populations. This study concluded that relative phospho-EGFR and phospho-ALK levels predict response to EGFR-TKIs and crizotinib, respectively [75]. Specifically, CA-EGFR/ALK tumors displayed diverse responses to first-line EGFR-TKIs based on baseline phospho-EGFR expression, whereas subsequent crizotinib efficacy correlated with phospho-ALK levels. Consequently, patients responsive to EGFR-TKIs typically exhibit high EGFR phosphorylation alongside low ALK phosphorylation, whereas the inverse profile favors crizotinib sensitivity. Conversely, Lou et al. identified elevated phospho-EGFR levels as a driver of crizotinib resistance, EGFR exon 20 T790M mutations, and histological transformation from L-ADC to SCLC [85]. Ultimately, how tumor heterogeneity encompassing gene mutation abundance and protein phosphorylation profiles impacts TKI efficacy demands deeper investigation.

The EGFR and ALK genes share a common signaling pathway, leading to reciprocal resistance mechanisms in NSCLC. Specifically, an ALK rearrangement can induce resistance to EGFR-TKIs in EGFR-mutated patients, while EGFR mutations and signaling activation drive ALK-TKI resistance [106]. As a result, tumor cells with both EGFR mutations and ALK rearrangements are expected to develop resistance to both EGFR-TKIs and ALK-TKIs over time. No agreed-upon guidelines currently exist for the best treatment approach for patients with CA-EGFR/ALK in NSCLC. Patients with both mutations are treated with EGFR-TKIs as a first-line treatment. After disease progression, ALK-TKIs are commonly administered. However, findings on the effectiveness of EGFR-TKIs and ALK-TKIs in these patients remain conflicting.

Many studies reported good responses to EGFR TKIs in cases of CA-EGFR/ALK [68,69], as well as excellent responses to ALK TKIs in cases of CA-EGFR/ALK [70,74,89]. Yang et al. reported an 80% PR (8 out of 10) in patients treated with first-line EGFR-TKIs, with a median PFS of 11.2 months [75]. Lou et al. found that 90% of patients with CA-EGFR/ALK responded well to first-line EGFR TKI [85]. Wang et al. showed that patients with CA-EGFR/ALK had similar ORRs after EGFR-TKI, but inferior PFS compared with those with an isolated EGFR mutation [69]. Ulivi et al. showed that among patients with EGFR mutations who received first-line therapy, 81.8% observed clinical benefit, compared with 67% in CA-EGFR/ALK [86]. Pooled data showed no statistical difference in DCR among cases receiving EGFR therapy, ALK therapy, or a combination of EGFR and ALK therapies. This difference was not significant with first-line or second-line therapy options.

Some studies showed a shorter PFS in CA-EGFR/ALK cases with EGFR therapy compared to those with only EGFR mutations [91,93]. Zhao et al. noted similar response rates and PFS between CA-EGFR/ALK and EGFR-only mutant cases with EGFR TKIs, as well as ALK TKIs [95]. Both first-generation EGFR-TKIs and crizotinib are effective in this patient population, supporting sequential treatment as a standard management option. Alternatively, data from Liu et al. suggest that dual-TKI therapy may be more practical and effective than single-TKI monotherapy for co-altered cases [97]. More empirical data is needed to evaluate the effectiveness of EGFR/ALK dual-TKIs as targeted drugs for NSCLC patients with both EGFR and ALK mutations. Most patients with CA-EGFR/ALK are treated with first-generation EGFR and ALK TKIs. More data with third-generation EGFR TKIs and newer-generation ALK TKIs, alone or in combination, would provide new insight into the management of CA-EGFR/ALK in NSCLC.

OS data are also conflicting. Kim et al. showed that patients harboring compound mutations had significantly shorter OS than those with simple mutations [84]. Lou et al. showed a statistically significant inferior OS in CA-EGFR/ALK cases compared to ALK-R cases [85]. Schmid et al. showed that patients with CA-EGFR/ALK may have worse outcomes with ALK and EGFR TKIs than those without either alteration [88]. Hu et al. reported no statistically significant difference in OS between patients harboring isolated EGFR mutations and those with concurrent genetic alterations [91]. Some studies have shown little impact on OS or PFS [92,93]. Zhao et al. demonstrated median OS times of 27.1 months for EGFR-mutant, 36.2 months for ALK-R, and 36.8 months for CA-EGFR/ALK cohorts [95]. The CA-EGFR/ALK subgroup tended to have a longer OS than the EGFR-mutant cohort, although this difference was not statistically significant (p = 0.12). Noronha et al. showed that at a median follow-up of 32 months, patients who received both EGFR- and ALK-directed therapies achieved a significantly longer median OS of 39.2 months, compared with 7.5 months for those who received only one or neither agent. Ultimately, ALK-directed therapy emerged as a significant independent predictor of prolonged OS [103].

Limitations

The study constitutes a retrospective analysis of published literature and is subject to various biases. We assessed data according to the defined proforma; however, many studies did not adhere to it, leaving demographic, clinicopathological, and therapeutic details missing for several variables. Consequently, the available cases for each variable are limited. Because many cases were still receiving treatment without progression or death at the time of publication, we could not calculate the median PFS or OS. Most studies used first-generation anti-EGFR TKIs and crizotinib, with osimertinib or alectinib used in only a small number of cases. The collective interpretation of the data is expected to inform the selection of anti-EGFR versus anti-ALK versus combined anti-EGFR and anti-ALK therapies. However, the decision to use newer-generation TKIs ultimately remains at the treating physicians' discretion.

## Conclusions

CA-EGFR/ALK cases are rare, with approximately 300 reported cases since the initial documentation in 2008. These instances predominantly manifest in non-smoking females with adenocarcinomas. Notably, there is a dearth of randomized or phase II studies addressing this sporadic molecular event explicitly. Available data primarily comprise case reports, small case series, and institutional analyses. The predominant treatment approach involves the administration of EGFR TKIs, followed by ALK TKIs, and, less frequently, a combination of both. However, the limited availability of frontline use data for third-generation oral TKIs and financial constraints may have influenced treatment decisions.

While response rates to both EGFR TKIs and ALK TKIs have generally been favorable, there are varied results and interpretations. Moreover, the reported PFS and OS outcomes exhibit inconsistencies across the published literature. Although a biological rationale favors the combination of EGFR and ALK TKIs, promising outcomes with acceptable toxicity profiles have been observed only in a small number of cases. Notably, there needs to be more consensus regarding the biomarkers that could guide the selection of optimal first-line TKIs. There exists a clear need for research to identify the preferred anti-EGFR or ALK TKI and the role of combination TKIs. A future clinical trial specifically designed for these patients is warranted to address these unmet needs.
